# Miscellaneous

**Published:** 1889-03

**Authors:** 


### MISCELLANEOUS.

THE fact that the elementary substances now number, according to chemists, full seventy, shows an increase, within the last fifty years, of nearly one-fourth in the number known. An atom of oxygen or nitrogen is said to have a diameter of one-ten-millionth part of a centimetre; they are supposed to be in a state of constant motion, at the rate of seventy miles a minute, and to make them visible, the present highest known magnifying power of the microscope would have to be increased nearly a thousand fold.

WARNER'S Log Cabin Remedies—old-fashioned, simple compounds, used in the days of our hardy forefathers, are "old timers," but "old reliable." They comprise: "WARNER'S LOG CABIN SARSAPARILLA," "Hops and Buchu Remedy," "Cough and Consumption Remedy," "Hair Tonic," "Extract," for External and Internal use, "Plasters," "Rose Cream," for Catarrh, and "Liver Pills." They are put up by H. H. Warner & Co., proprietors of Warner's Safe Remedies, and promise to equal the standard value of those great preparations. All druggists keep them.

SOME months ago, while Dr. G. W. Galvin, of Boston, was attending Mrs. Mary Parker and her new-born child, a bulldog, a great pet in the family, got into the room, and jealous of the attentions paid the little one, attempted to bite it. The Doctor successfully defended the baby and got it out of the room in the arms of the nurse, but then the dog attacked him, and before he could subdue it, the vicious brute bit him so that he was confined to the house for some days. The Doctor, when he recovered, wanted the mother whose infant he had saved to pay him for the time lost on account of the bites from her dog, but could get no compensation until he appealed to the law. He has just obtained a verdict of $700 damages against Mrs. Parker.

A GENTLEMAN attached to a foreign legation, who has seen a good deal of New York city this year, remarked recently that he was continually surprised at the number of vacuous and pompous looking youths who crowd the doorways at public balls and receptions. "I never see them anywhere else," he said, "and apparently they live in evening dress. There are eighty or a hundred of them, and they all seem precisely alike. They have narrow shoulders, slim figures, and some of them affect the single glass. They crowd around the doorways and stare stupidly at the women without making the faintest effort to join the spirit of the occasion or render themselves useful to their hostess. Further than this, they seem so remarkably ill-bred. A dozen times this winter I have rather made an ass of myself by presuming to address these sons of vacuity. When I chanced to be among them in a doorway or was moved by some unconscious impulse, I ventured to speak just as a man would in society anywhere else in the world to my nearest neighbor. I was invariably regaled by a stare, and an answer which would be a stretch of truth to be called civil. It is not to be wondered at that American young men are so unpopular abroad. One can afford to be snubbed by a duke, but nobody will swallow an insult from a man who has not social position, wealth or wit to recommend him."—*N. Y. Sun.*

Bees and Ants.—A noteworthy case of division of labor is furnished by these insects. It is well known that a colony of bees or ants consists of at least three classes of individuals or "castes," males, females and workers. To the males and females belong exclusively the duties connected with bringing forth new generations of their kind; to the workers falls the task of building the nest and providing food. In some species of ants there is a fourth order called "soldiers." To these is relegated the duty of defending the colony against the attacks of their enemies. Here is as perfect an exemplification of the principle of division of labor as can be found in human society. The division of employment is less minute, but the fitness for this special task in these insect groups is far more perfect than that of the most adept craftsmen to their special occupations. For the insects have undergone bodily modification to fit them for their special duties. For example, in worker bees, parts that are homologous to the reproductive organs in the males, serve as stings; it can scarcely be doubted that here has been a modification of copulatory structures into organs of attack and defense. In these insects, therefore, specialization has not only taken place in respect to skill in action depending upon powers of intelligence (instincts) but also in respect to bodily structure.

Hypocrisy is a sort of homage vice pays to virtue. Drive out the vicious blood with Warner's Log Cabin Sarsaparilla, renew it with new and virtuous blood and there will be no hypocrisy in your strong and healthy nature. Best blood remedy and cheapest. All druggists sell it.

Formation of Coal.—The ultimate products of the decomposition of vegetable matter in the air are carbonic acid and water, both of which are absorbed by the atmosphere; so that practically nothing remains behind to tell the tale. When, however, vegetable matter is covered by water, only a partial decomposition results, and a variety of products are formed by the chemical changes which take place. This subaqueous decomposition with the chemical changes attendant upon it has been the principal agency in the formation of the several kinds of coal.

